# Characterization of Mild Acid Stress Response in an Engineered Acid-Tolerant *Escherichia coli* Strain

**DOI:** 10.3390/microorganisms12081565

**Published:** 2024-07-31

**Authors:** Jingliang Qin, Han Guo, Xiaoxue Wu, Shuai Ma, Xin Zhang, Xiaofeng Yang, Bin Liu, Lu Feng, Huanhuan Liu, Di Huang

**Affiliations:** 1 Tianjin Key Laboratory of Microbial Functional Genomics, TEDA Institute of Biological Sciences and Biotechnology, Nankai University, Tianjin 300457, China; qinjingliang@mail.nankai.edu.cn (J.Q.); liubin1981@nankai.edu.cn (B.L.);; 2 School of Biology and Biological Engineering, South China University of Technology, 382 East Outer Loop Road, University Park, Guangzhou 510006, China; 202020148595@mail.scut.edu.cn (X.Z.); biyangxf@scut.edu.cn (X.Y.); 3 Nankai International Advanced Research Institute, Nankai University, Shenzhen 518000, China; 4 State Key Laboratory of Food Nutrition and Safety, Tianjin University of Science & Technology, Tianjin 300457, China

**Keywords:** *Escherichia coli*, weighted gene coexpression network analysis, synthetic acid-tolerance module, mild acid stress, RNA sequencing

## Abstract

Engineering acid-tolerant microbial strains is a cost-effective approach to overcoming acid stress during industrial fermentation. We previously constructed an acid-tolerant strain (*Escherichia coli* SC3124) with enhanced growth robustness and productivity under mildly acidic conditions by fine-tuning the expression of synthetic acid-tolerance module genes consisting of a proton-consuming acid resistance system (*gad*E), a periplasmic chaperone (*hde*B), and ROS scavengers (*sod*B, *kat*E). However, the precise acid-tolerance mechanism of *E. coli* SC3124 remained unclear. In this study, the growth of *E. coli* SC3124 under mild acid stress (pH 6.0) was determined. The final OD600 of *E. coli* SC3124 at pH 6.0 was 131% and 124% of that of the parent *E. coli* MG1655 at pH 6.8 and pH 6.0, respectively. Transcriptome analysis revealed the significant upregulation of the genes involved in oxidative phosphorylation, the tricarboxylic acid (TCA) cycle, and lysine-dependent acid-resistance system in *E. coli* SC3124 at pH 6.0. Subsequently, a weighted gene coexpression network analysis was performed to systematically determine the metabolic perturbations of *E. coli* SC3124 with mild acid treatment, and we extracted the gene modules highly associated with different acid traits. The results showed two biologically significant coexpression modules, and 263 hub genes were identified. Specifically, the genes involved in ATP-binding cassette (ABC) transporters, oxidative phosphorylation, the TCA cycle, amino acid metabolism, and purine metabolism were highly positively associated with mild acid stress responses. We propose that the overexpression of synthetic acid-tolerance genes leads to metabolic changes that confer mild acid stress resistance in *E. coli*. Integrated omics platforms provide valuable information for understanding the regulatory mechanisms of mild acid tolerance in *E. coli* and highlight the important roles of oxidative phosphorylation and ABC transporters in mild acid stress regulation. These findings offer novel insights to better the design of acid-tolerant chasses to synthesize value-added chemicals in a green and sustainable manner.

## 1. Introduction

During industrial fermentation, microbes encounter acid stress due to the accumulation of acidic metabolites in the fermentation medium [1]. Acid stress increases intracellular microbial acidity, resulting in DNA damage and enzyme denaturation, ultimately leading to reduced microbial growth and fermentation yield [2,3,4]. Although acid stress can be mitigated by neutralizing the fermentation broth with a base, employing acid-tolerant microbial strains offers a more efficient and cost-effective solution [3,4,5].

Acid stress tolerance in microorganisms is a complex polygenic trait that cannot be achieved by overexpressing a single functional gene [1]. For example, in *Escherichia coli* (*E. coli*), there are variable acidic stress response mechanisms that include metabolic, physiological, and proton-consuming acid resistance (AR) systems [4]. Many strategies have been developed to increase the acid tolerance of *E. coli* [2]. The most utilized method is the genome-wide engineering strategy, known as global transcription machinery engineering; it involves the molecular engineering of global transcription regulators such as the global regulator cAMP receptor protein (CRP), histone-like nucleoid structuring factor (H-NS), exogenous global regulator (IrrE), and global regulator sigma D factor (RpoD) [6,7,8,9]. Although effective, this strategy may simultaneously result in the perturbation of hundreds of nonessential genes in *E. coli* cells, ultimately causing the inefficient consumption of cellular energy and resources. Recently, the assembly of synthetic stress-tolerance modules via the combinatorial expression of multiple stress-tolerance genes was shown to enhance cellular stress tolerance [10]. The overexpressions of the DNA-binding protein gene *hu* (involved in DNA protection), RNA-binding protein gene *rbp* (related to RNA protection), and ATP-dependent serine protease gene *clpP* (involved in misfolded protein degradation) in *E. coli* DH10B increased its survival rate by more than 600-fold during acid shock at pH 1.9 [11].

A large majority of the studies on acid tolerance have mainly focused on cell survival under extreme pH conditions. However, enhanced productivity and growth under mildly and moderately acidic conditions are more valuable for industrial applications because these conditions mimic the natural or engineered environments where microorganisms are harnessed for commercial purposes, such as fermentation, bioremediation, and industrial enzyme production. *E. coli* has developed an acid resistance system to extreme acid stress (external pH of 2–3, internal pH~4.5) and an acid tolerance response system towards mild and moderate acid stress (external pH of 4–5, internal pH~7.0) [4]. Mild acid stress can also be defined as an external pH range of 5–6 and an internal pH of ~7.4 [12]. Periplasmic chaperones, proton-consuming AR systems, and reactive oxygen species (ROS) scavengers are the three most important contributors to the acid-tolerant phenotype of *E. coli* at mild and moderate acidic pH (4.5) [6,7]. However, hundreds of genes are involved in acid and oxidative stress regulation in *E. coli* [9,13], which makes it difficult to develop reasonable stress-tolerance modules.

In a previous study, we designed a set of synthetic acid-tolerance modules to improve the acid tolerance of *E. coli* under mildly acidic conditions (external pH of 5–6) by fine-tuning the expression of genes involved in proton-consuming AR systems (*gad*E), periplasmic chaperones (*hde*B), and ROS scavenging (*sod*B and *kat*E) [14]. The transcriptional regulator, GadE, is a key activator of the proton-consuming AR2 system [15]. The periplasmic chaperones, HdeA and HdeB, prevent periplasmic protein aggregation at low pH, with HdeB being more efficient under mild acid stress (pH 4–6) [16]. Superoxide dismutase (SodB) and catalase (KatE) catalyze the conversion of superoxide radicals to hydrogen peroxide, which is further converted into water and oxygen [17,18]. Using stepwise screening, we obtained a series of synthetic acid-tolerance modules with improved growth and productivity under mildly acidic conditions (pH 6.0). Among them, overexpression of the most efficient module (SC3124) in the lysine-producing *E. coli* MG1655 SCEcL3 strain resulted in an increased lysine yield, up to 115% and 118%, compared with that by the parent *E. coli* MG1655 in 1.3 L bioreactors at pH 6.0, and 6.8, respectively [14]. By rough estimation, this strategy could save approximately 260 kg of glucose per ton of lysine produced, while fermentation at pH 6.0 could reduce the amount of acid added during industrial lysine purification by 5–10% [14]. Thus, we aimed to investigate the effects of SC3124 overexpression in *E. coli* under different culture times and different acidic conditions, especially under mild acid stress.

Omics technology provides a novel opportunity to understand gene changes and the potential metabolic response mechanism in *E. coli* under various conditions. Traditional transcriptome analysis methods such as differential gene expression analysis can be used to compare the differentially expressed genes between two groups and to obtain significant pathway enrichments, targeting differentially expressed genes. Weighted gene coexpression network analysis (WGCNA) is often used to investigate the complex associations between phenotypes and genes [19]. WGCNA transforms gene expression data into co-expression modules that provide insights into the signaling networks that potentially lead to phenotypic traits. This approach facilitates the analysis of the dynamic expression of gene modules or genes associated with phenotypes, allowing us to obtain significant modules, identify key pathways, and evaluate potential regulation in *E. coli* under mild acid stress [20].

We used an integrated strategy, based on RNA sequencing (RNA-seq) and weighted gene co-expression network analysis, to analyze the transcriptomic and metabolic responses of *E. coli* MG1655 harboring SC3124 to mild acid stress and compared these responses to those of the parent *E. coli* MG1655.

## 2. Materials and Methods

### 2.1. Strains and Culture Conditions

Unless otherwise stated, *E. coli* MG1655 and its derivative strains were propagated in LB broth (10 g/L peptone, 5 g/L yeast extract, 10 g/L NaCl) supplemented with 20 g/L glucose (LBG medium). Chloramphenicol (34 μg/mL) was supplemented into the cultures when necessary. For growth curve determination and transcriptomic analysis, *E. coli* SC3124 and *E. coli* MG1655 carrying empty pACYC184 vector were grown in 40 mL of LBG broth (initial pH 6.8) for 16 h at 37 °C with the appropriate chloramphenicol. Subsequently, they were subcultured in a parallel bioreactor (T&J Bio-engineering Co., Ltd., Shanghai, China), with a diameter of 97 mm, a sterilization height of 176 mm, and a total capacity of 1.3 L. Each bioreactor contained 360 mL of fresh LBG medium with appropriate chloramphenicol for 24 h at 37 °C. The pH was adjusted to 6.0 or 6.8 by adding 1M HCl or ammonia water (25% *w/v*). The dissolved oxygen was monitored online and was maintained between ~40 and 60% by adjusting the agitation rate from 200 to 1000 rpm with an aeration rate of 0.5 air volume/culture volume/min (vvm). Cell growth was quantitatively assessed via OD600 measurements using an ultraviolet spectrophotometer at 600 nm with a standard 1 cm light transmission length. When OD600 reached a value of 1 under these conditions, the empirical correlation between OD600 and the bacterial suspension concentration indicated that OD600 = 1 corresponded to a bacterial suspension concentration of approximately 8 × 10^8^ CFU/mL.

### 2.2. RNA-Seq Quantification of Gene Expression Levels

*E. coli* cells grown under mildly acidic (pH 6.0) and neutral (pH 6.8) conditions were harvested at 6 and 24 h, respectively, and used for transcriptomic analysis. All eight groups of samples obtained were subjected to RNA-seq including SC3124-pH 6.0-6 h, SC3124-pH 6.0-24 h, SC3124-pH 6.8-6 h, SC3124-pH 6.8-24 h, WT-pH 6.0-6 h, WT-pH 6.0-24 h, WT-pH 6.8-6 h, and WT-pH 6.8-24 h. For each group, three biological replicates were used in the transcriptomic analysis. RNA was extracted from each sample using TRIzol Reagent (Invitrogen, Waltham, MA, USA). The genomic DNA was digested using DNase I (Takara Bio, Shiga, Japan), following the manufacturer’s instructions. Sequencing libraries were constructed using NEBNext@ Ultra™ Directional RNA Library Prep Kit for Illumina (New England Biolabs Ltd., Ipswich, MA, USA), following the manufacturer’s instructions, and index codes were added to the attribute sequences of each sample. The index-coded samples were clustered on a cBot Cluster Generation System using a TruSeq PE Cluster Kit v3-cBot-HS (Illumina, San Diego, CA, USA). The libraries were sequenced using the Illumina NovaSeq 6000 platform to generate paired-end reads. The raw data were filtered to remove adapter sequences, reads containing poly-N tracts, and low-quality sequence reads with Q-value ≤ 20. Next, the remaining high-quality reads were mapped to the *E. coli* K12 substrain MG1655 reference genome (NC_000913.3) using Bowtie 2 v2.2.3 [21]. The number of reads mapped to each gene was counted using HTSeq v0.6.1 [22]. The expression value of each gene was determined by calculating the number of fragments per kilobase of transcript sequence per million base pairs sequenced (FPKM) based on the length and read counts mapped to each gene. Differential expression analysis was performed using DESeq2 v1.38.3 [23]. Genes were considered differentially expressed in SC3124 when compared to WT at a *q*-value (adjusted *p*-value) < 0.05 and |log2(fold-change)| > 1 [24]. Differentially expressed genes were clustered by the relative expression level of FPKM using the Euclidean distance.

### 2.3. Construction of the WCGNA Network

The WGCNA model was constructed using the WGCNA package online tutorial in R by calculating the weighted Pearson correlation matrices relative to FPKM [25]. Gene expression correlation coefficients were calculated to identify a suitable soft threshold for gene network construction based on the scale-free topology criterion. A gradient method was used to evaluate the scale-free fit index and mean degree of connectivity between different coexpression modules with power values ranging from 1 to 20. Using TOM-based module dissimilarity (1-TOM) analysis, genes with highly similar correlation relationships were grouped into the same modules through hierarchical clustering. Each gene module was assigned a color, with genes not sorted into any specific module grouped in grey. The minimum number of genes in each module was set to 30 to ensure the high reliability of the results. Pearson correlation coefficients between the eigengene module and each trait (OD600, SC3124, WT, pH6.0, and pH6.8) were calculated to identify the modules highly correlated with the synthetic acid tolerance module and different pH conditions. The GS and MM were also calculated. Genes in the modules with significant module–trait associations (coefficient > 0.55 and *p* ≤ 0.01) were included in the functional enrichment analysis.

### 2.4. Hub Gene Determination

Genes with high significance for each trait and high MMs in the modules of interest were characterized using GS and MM [26]. The intramodular connectivity of each gene was determined by dividing the sum of the strengths of its connections with other module genes by the maximum intramodular connectivity. Genes with maximum intramodular connectivity were considered intramodular hub genes (GS > 0.5; MM > 0.5).

### 2.5. GO and KEGG Enrichment Analysis

GO and KEGG enrichment analyses of the hub genes were performed using KOBAS v3.0 [27] (http://bioinfo.org/kobas, accessed on 2 October 2023) and TBtools v2.012 [28] software. Enriched terms with a Bonferroni-corrected *p*-value < 0.05 were regarded as significant and subjected to biological function annotation.

### 2.6. PPI Analysis

The STRING (https://cn.string-db.org/, accessed on 7 October 2023) [29] online database was used for PPI analysis using the default parameters. The gene interaction network was visualized using Cytoscape v3.9.1 [30]. A Cytoscape-plugin Molecular Complex Detection (MCODE) [31] was used to extract the core subnetworks, with a K-core value ≥ 2, which is a Cytoscape plug-in allowing the detection of densely connected regions in large PPI networks that likely represent molecular complexes.

### 2.7. Data Visualization

Data generated from RNA sequencing analyses were visualized with R v4.2.2 (https://www.R-project.org/, accessed on 1 August 2023) using the following packages: ggplot2 for volcano plots and WGCNA and flashClust for WGCNA analysis. FPKM heatmap, GO term, and KEGG pathway plots were generated using ChiPlot (https://www.chiplot.online/, accessed on 20 November 2023).

## 3. Results

### 3.1. Growth and Transcriptome Changes under Mild Acid Stress

**Figure 1:**
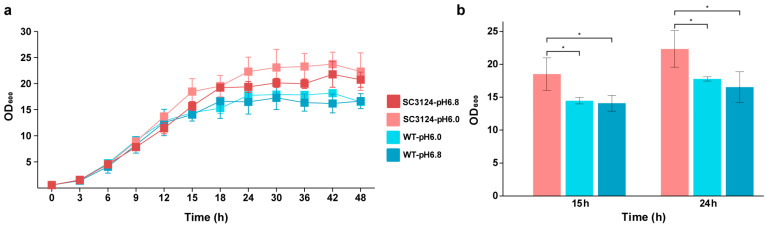
Growth of *E. coli* strains in 1.3 L parallel bioreactor under different pH conditions. (**a**) Growth curves of *E. coli* strains under different pH conditions. *E. coli* MG1655 carrying synthetic acid-tolerance module SC3124, and *E. coli* MG1655 are represented as SC3124 and WT, respectively. (**b**) The results of the statistical analysis of *E. coli* growth under different pH conditions at 15 h and 24 h. Each experiment was performed in three biological replicates. * Represents significance (*p* < 0.05).

The growth curves of *E. coli* MG1655 harboring the synthetic acid-tolerance module SC3124 [14] (designated as *E. coli* SC3124) and of the wild-type *E. coli* MG1655 (WT) carrying empty pACYC184 vector at pH 6.0 and 6.8 were measured. At 15 h, the OD600 of *E. coli* SC3124 at pH 6.0 was higher than that of the WT (at pH 6.0 and pH 6.8, *p* < 0.05). At 24 h, the final OD600 of *E. coli* SC3124 at pH 6.0 reached 22.3, which was 131% and 126% of that of the WT grown at pH 6.8 and pH 6.0 (*p* < 0.05), respectively (Figure 1a,b). The results indicated that the overexpression of the module SC3124 in *E. coli* significantly increased its growth under mildly acidic and neutral conditions, which is consistent with previous study results [14].

To explore the mild acid-stress tolerance mechanism, SC3124 and WT *E. coli* were grown in mildly acidic (pH 6.0) and neutral (pH 6.8) conditions, and the cultures were harvested in the exponential (6 h) and stationary (24 h) state, respectively. A total of eight groups of samples (SC3124-pH 6.0-6 h, SC3124-pH 6.0-24 h, SC3124-pH 6.8-6 h, SC3124-pH 6.8-24 h, WT-pH 6.0-6 h, WT-pH 6.0-24 h, WT-pH 6.8-6 h, and WT-pH 6.8-24 h) were subjected to transcriptome analysis using Illumina RNA sequencing technology. The dataset of each sample had an average size of 3 Gb, with raw reads ranging from 7.18 to 13.14 million and clean reads from 7.15 to 13.10 million.

### 3.2. Differential Gene Expression Analysis

**Figure 2:**
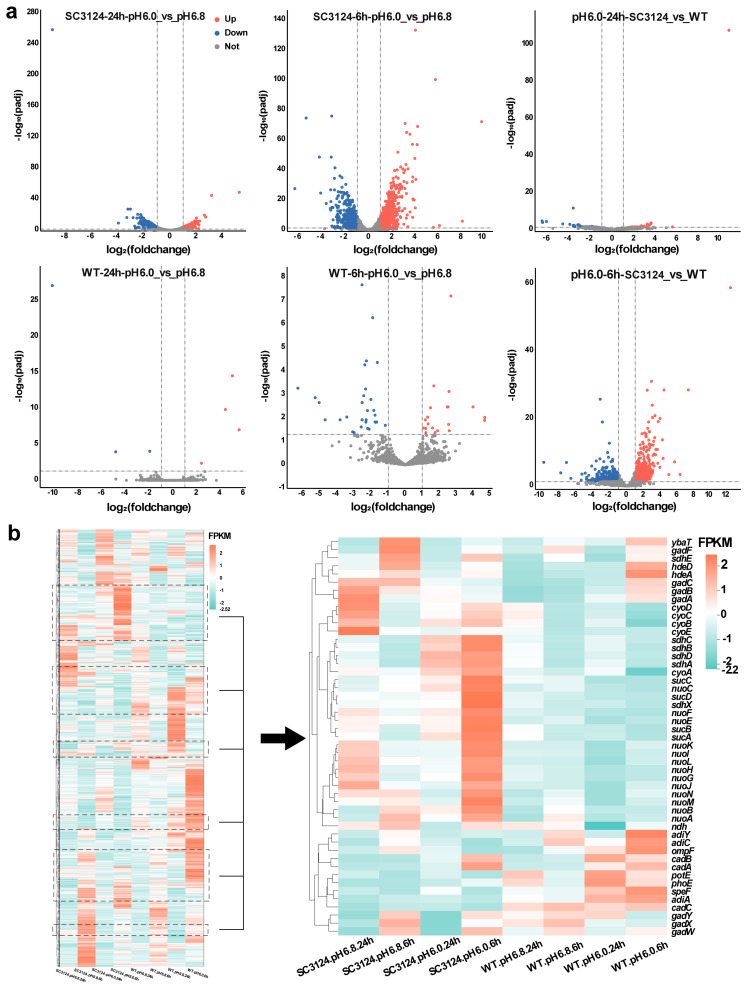
Expression analysis of the *E. coli* MG1655 and SC3124 grown at different pH. (**a**) Volcano plots of differentially expressed genes in different groups. The horizontal axis represents multiple logarithmic changes in gene expression, and the vertical axis represents statistically significant changes in gene expression (negative logarithm). Blue dots indicate downregulated genes, red dots indicate upregulated genes, and grey dots indicate genes not differentially expressed. Dashed lines represent thresholds for statistical significance or the magnitude of change. (**b**) Clustering heatmap of differential gene expression. Color from red to blue indicates FPKM from large to small. The dashed boxes represent the relative locations of the genes. The right panel shows the heatmap of the genes marked with dashed boxes in the left panel. *E. coli* SC3124 and *E. coli* MG1655 are represented as SC3124 and WT, respectively. Three biological replicates of each group were analyzed.

Differential gene expression analysis was performed using DESeq2 software v1.38.3 [23] and R Studio v4.2.2. For the samples with three biological replicates, genes with a *q*-value (adjusted *p*-value) < 0.05 and |log2(foldchange)| > 1 were considered differentially expressed and were visualized using a volcano plot (Figure 2a). Specifically, at pH 6.0, *E. coli* SC3124 displayed a robust response, with 571 and 208 genes upregulated and 525 and 310 genes downregulated at 6 and 24 h, respectively, compared to the neutral pH 6.8 condition. In contrast, the WT strain showed a more muted response, with only 20 and 4 genes upregulated and 30 and 3 genes downregulated under the same acidic conditions at 6 and 24 h. When comparing SC3124 to the WT at pH 6.0, we observed a significant reshuffling of gene expression, with 499 and 35 genes downregulated and 481 and 91 genes upregulated in SC3124 at 6 and 24 h, respectively. These results showed that mild acid treatment resulted in more significant differences in gene expression in *E. coli* SC3124.

To evaluate the differential gene expression patterns of SC3124 and the WT, clustering analysis was performed on the differentially expressed genes (Figure 2b). Different-colored regions represent different clustering information. Genes with similar colors have similar expression patterns. As shown in Figure 2b, the differential gene expression patterns of SC3124 differed from those of the WT when grown in an acidic environment. In SC3124 at pH 6.0, the expressions of *cyo*, *ndh*, *nuo*, and *sdh* genes, encoding cytochrome *bo* oxidase, NADH dehydrogenase II, NADH dehydrogenase I, and succinate dehydrogenase, composed of the electron transport chain during aerobic respiration, and *suc* genes encoding 2-oxoglutarate decarboxylase and succinyl-CoA synthetase, the key enzymes of the tricarboxylic acid (TCA) cycle, were significantly upregulated (by more than three-fold) at 6 and 24 h compared with those at pH 6.8. In addition, the lysine-dependent AR (LDAR) system gene, *cad*AB, was upregulated in SC3124 at 6 h (pH 6.0). Compared with pH 6.8, in the WT, the genes involved in four amino-acid-dependent AR systems, including glutamic-acid-dependent AR (GDAR, *gad*ABC, *yba*T), arginine-dependent AR (ADAR, *adi*ACY), LDAR (*cad*ABC), and ornithine-dependent AR (ODAR, *spe*F, *pot*E) systems, as well as outer membrane porin genes (*pho*E, *omp*F) capable of reducing proton influx in the low pH environment, and chaperone protein genes (*hde*A) managing the effects of acid damage in enzyme proteins, were notably upregulated under pH 6.0 at 6 h, while most were downregulated at 24 h. These results indicated different responses between *E. coli* SC3124 and the WT to mild acid stress.

### 3.3. WGCNA Analysis

#### 3.3.1. Soft Threshold Determination, Network Topology Analysis of Adjacency Matrices Based on WGCNA

**Figure 3:**
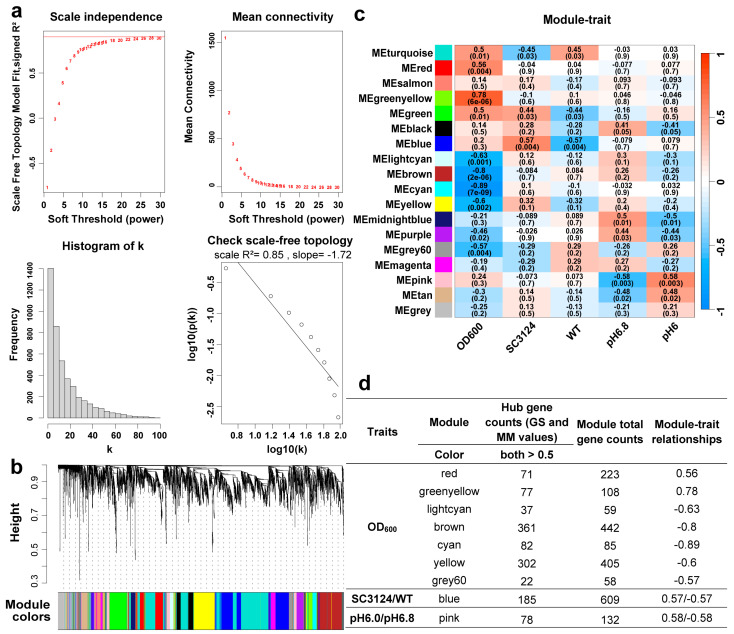
WGCNA of all expressed genes. (**a**) Network topology analysis for the soft threshold powers of adjacency matrices. Red numbers indicate the soft-threshold power corresponding to the correlation coefficient square value and mean connectivity. The correlation coefficient of the linear relationship between the logarithm of the gene connectivity (k) and the logarithm of the proportion of genes exhibiting that connectivity (p(k)) was derived from the analysis of individual adjacency matrices. Here, k represents the degree of connectivity a gene has within the network, and p(k) denotes the relative frequency of genes that possess connectivity k. (**b**) Hierarchical clustering tree (clustering dendrogram) of all expressed genes. Each leaf of the tree corresponds to one gene. The major tree branches constitute 18 modules, labeled with different colors. (**c**) Module–trait relationships. The row corresponds to the module and the column corresponds to the trait. The SC3124 and WT traits for *E. coli* MG1655, carrying the synthetic acid-tolerance module, and the wild-type *E. coli* MG1655, carrying empty pACYC184 vector, respectively. Modules are colored as shown in the legend. Positive and negative correlation is presented by blue and red, respectively. The grey module represents a collection of genes that could not be grouped into other modules. The value in each cell represents the correlation coefficient between the module and the trait. The *p*-value is shown in parentheses in each cell. “ME” stands for “module eigengene,” which is a composite measure of gene expression within a module. (**d**) Summary of the relationships between traits and WGCNA modules. WGCNA modules that were highly associated with traits (correction coefficient > 0.55 and *p* ≤ 0.01) and the gene counts in each module. + represents positive correlation and − represents negative correlation.

To investigate the relationship between the gene expression profiles and acid stress tolerance traits and to identify highly synergistic gene sets as well as candidate biomarker genes or metabolic targets, a WGCNA model was constructed using transcriptomic data according to gene set connectivity. A total of 4392 genes were used for the weighted gene coexpression network construction. The optimal power value was 16 when the scale-free fit index was >0.8 (Figure 3a), which met the requirement for WGCNA modeling.

#### 3.3.2. Gene Clustering and Module–Trait Relationships

The clustering dendrogram of all the expressed genes is shown in Figure 3b. Based on hierarchical clustering and dynamic tree cutting using the topological overlap measure (TOM), 4392 candidate genes were clustered into 18 modules, each marked with a different color (Table S1). We analyzed the module–trait relationships using correlations between the module eigengenes and acid traits (OD600, SC3124, WT, pH 6.0, and pH 6.8) to identify coexpression modules with significant correlations with the synthetic acid-tolerance module and different pH conditions. The WGCNA modules with correction coefficients > 0.55 and *p* ≤ 0.01 were considered as highly associated with acid-tolerance traits, and a total of 11 module–trait relationships were identified (Figure 3c).

For each gene expression profile, gene significance (GS) was calculated as the absolute value of the correlation between the expression profile and each external trait, and module membership (MM) was defined as the correlation between the expression profile and each module eigengene. The calculation of the GS and MM values allowed for the identification of modules of interest through the selection of genes highly significant for each trait that had high MMs. The scatter plots of GS vs. MM for each module are shown in Figure S1. Additional details on the GS and MM are provided in Figure S1. GS and MM were correlated, indicating that genes significantly associated with acid-tolerance traits were also important elements of the modules.

Hub genes within modules are likely critical and representative of the module’s function in a network. Genes with GS and MM values > 0.5 were defined as hub genes in their respective modules. The WGCNA modules, related traits, eigengene counts, and hub genes are summarized in Figure 3d. The red and green-yellow modules were highly positively correlated with the OD600 trait, whereas the brown, cyan, yellow, and grey60 modules were highly negatively correlated with the OD600 trait. Only the blue module was highly associated with the SC3124 and WT traits, but the opposite trend was observed. Similarly, only the pink module was highly positively and negatively correlated to the pH 6.0 and pH 6.8 trait, respectively.

#### 3.3.3. Functional Enrichment Analysis of Hub Genes Highly Correlated with Traits

**Figure 4:**
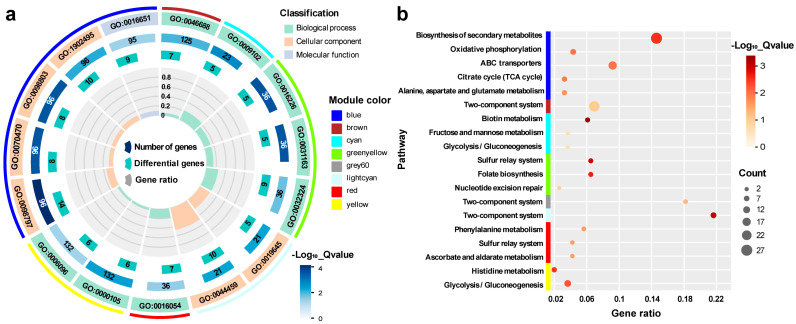
GO (**a**) and KEGG (**b**) enrichment analysis of the hub genes. The enriched items with FDR < 0.05 were acceptable.

The hub genes in each WGCNA module that were highly associated with acid traits were subjected to gene ontology (GO) and Kyoto Encyclopedia of Genes and Genome (KEGG) pathway enrichment analyses using KOBAS [27] and TBtools [28] (Figure 4).

### 3.4. OD600

The enriched GO terms for the red and green-yellow modules that were highly positively associated with the OD600 trait were organic acid catabolic process (GO:0016054), iron-sulfur cluster assembly (GO:0016226), metallosulfur cluster assembly (GO:0031163), and molybdopterin cofactor biosynthetic process (GO:0032324) (Figure 4a). The enriched KEGG pathways for the two modules were ascorbate and aldarate metabolism (eco00053), phenylalanine metabolism (eco00360), the sulfur relay system (eco04122), nucleotide excision repair (eco03420), and folate biosynthesis (eco00790). In the lightcyan, brown, cyan, yellow, and grey60 modules, which were negatively associated with the OD600 trait, the enriched GO terms were anaerobic respiration (GO:0009061), response to copper ion (GO:0046688), biotin biosynthetic process (GO:0009102), histidine biosynthetic process (GO:0000105), obsolete plasma membrane part (GO:0044459), and glycolytic process (GO:0006096); and the enriched KEGG pathways included the two-component system (eco02020), biotin metabolism (eco00780), fructose and mannose metabolism (eco00051), histidine metabolism (eco00340), and glycolysis/gluconeogenesis (eco00010) (Figure 4b).

### 3.5. SC3124 and WT

The SC3124 trait was highly positively associated with the blue module, whereas the opposite trend was observed for the WT trait. In this module, six GO terms were enriched (GO:0098797, plasma membrane protein complex; GO:0098803, respiratory chain complex; GO:0070470, plasma membrane respirasome; GO:1902495, transmembrane transporter complex; GO:1990204, oxidoreductase complex; and GO:0016651, oxidoreductase activity acting on NADH or NADPH) (Figure 4a), and eight KEGG terms were enriched [eco00190, oxidative phosphorylation; eco02010, ATP-binding cassette (ABC) transporters; eco00020, citrate cycle; eco01110, alanine, aspartate, and glutamate metabolism; and eco00250, biosynthesis of secondary metabolites] (Figure 4b). These results indicated that the overexpression of the synthetic acid-tolerance module resulted in metabolic disturbances. As there was only one synthetic acid-tolerance module difference between the two groups of strains, the gene sets significantly associated with *E. coli* SC3124 and WT were potential targets for the synthetic acid-tolerance module to function.

### 3.6. pH 6.0 and pH 6.8

The pH 6.0 trait was highly positively correlated with the pink module. In contrast, the pH 6.8 trait was highly negatively correlated with this module (Figure 3d). This indicated that mild acid treatment affected gene expression in *E. coli* SC3124 and the WT. Neither the GO nor KEGG pathways were enriched in the pink module, suggesting that the hub genes of the pink module were relatively dispersed in the metabolic system and were difficult to enrich.

### 3.7. PPI Analysis of Hub Genes in Blue and Pink Modules

**Figure 5:**
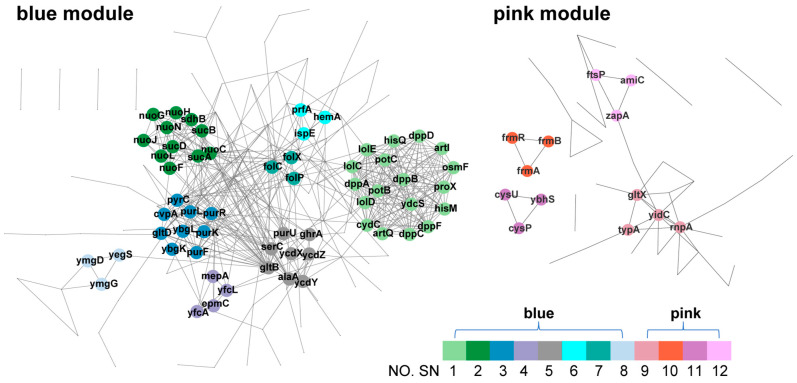
Protein–protein interactions between hub genes. PPI networks of genes from blue and pink modules. The blue module PPI network represents the interactions within the hub gene of the module correlated with either SC3124 or WT. In contrast, the pink module PPI network shows the interactions within the hub gene of the module correlated with either pH 6.0 or pH 6.8. Each network contains hub genes from highly associated WGCNA modules. Colors and numbers represent different subnetworks extracted by MCODE. Network nodes K-core value < 2 are hidden.

Protein–protein interaction (PPI) analysis was performed to deeply explore the internal characteristics of the interesting gene modules. We focused on the differential expression of the genes between *E. coli* SC3124 and WT under acidic and neutral pH conditions. Thus, the hub genes of the blue module, which was highly associated with SC3124/WT traits, and the pink module, which was highly associated with pH 6.0/pH 6.8 traits, were employed for PPI analysis. The predicted functional associations between hub proteins and each trait were identified using the STRING database based on known interactions, predicted interactions, and other evidence (text mining, coexpression, and protein homology). Twelve subnetworks (SNs) of high connectivity revealed the existence of highly interconnected gene sets (Figure 5, Table 1 and Table 2). The genes in each subnetwork were enriched with the GO and KEGG databases, and the items with a minimum FDR (<0.05) are listed in Table 1 and Table 2.

A total of 185 hub genes from the blue module of the WGCNA were used to construct the PPI network. Eight dense regions (SN-1 to 8; Figure 5 and Table 1) were extracted. SN-1 contained 18 genes encoding the components of ABC transporters (*dpp*ABCD and *dpp*F, dipeptide ABC transporter system; *pro*X and *osm*F, glycine betaine ABC transporter periplasmic binding proteins; *his*M and *his*Q, membrane components of lysine/arginine/ornithine ABC transporter and histidine ABC transporter; *art*I and *art*Q, L-arginine ABC transporter periplasmic binding proteins; *cyd*C, glutathione/L-cysteine ABC exporter subunit; *pot*BC, membrane subunits of spermidine preferential ABC transporter; *lol*CDE, ABC transporter involved in lipoprotein trafficking; *ydc*S, putative ABC transporter), suggesting that microbial cells need to import more nutrients or other molecules as well as export toxins or lipids across the membrane to protect the cells against acid stress. SN-2 contained 11 genes enriched in the oxidative phosphorylation and TCA cycle pathways. Further analysis showed that *nuo*CFGHJLN encode NADH: quinone oxidoreductase subunits and *sdh*B encodes succinate: quinone oxidoreductase subunits, which are components of the electron transport chain. *suc*A and *suc*CD, which encode 2-oxoglutarate decarboxylase and succinyl-CoA synthetase subunits, respectively, and *sdh*B are involved in the TCA pathway. The upregulation of the components of the electron transport chain in *E. coli* SC3124 leads to a higher proton export rate, which confers tolerability to a drop in cytoplasmic pH via direct proton export. The genes enriched in SN-3, SN-5, and SN-7 were associated with alanine biosynthesis (*ala*A, glutamate-pyruvate aminotransferase), glutamate biosynthesis (*glt*B and *glt*D, glutamate synthase subunits), glutathione metabolism (*pxp*A and *pxp*C, 5-oxoprolinase components A and C), glycine, serine, and threonine metabolism (*ser*C, phosphoserine/phosphohydroxythreonine aminotransferase, *ghr*A, glyoxylate/hydroxypyruvate reductase A), folate biosynthesis (*fol*C and *fol*P, dihydrofolate synthetase, *fol*X, dihydroneopterin triphosphate 2’-epimerase), purine biosynthesis (*pur*FKLRU, part of purine nucleotide biosynthesis operon), and pyrimidine biosynthesis (*pyr*C, dihydroorotase). This indicated that the microbes required nutrient and proteome resources to perform metabolic and cellular processes under acidic conditions. SN-4, SN-6, and SN-8 contained ten genes altogether. However, no GO terms or KEGG pathways were enriched.

In the pink module, 78 hub genes were used to construct the PPI network, and four dense regions with high internal connectivity (SN-9–SN-12; Figure 5, Table 2) were identified. Genes enriched in SN-9 and SN-12 are involved in the insertion and proper folding of inner membrane proteins (*yid*C, membrane protein insertase), tRNA precursor processing (*rnp*A, ribonuclease P protein component; *glt*X, glutamate-tRNA ligase), ribosome assembly (*bip*A, 50S ribosomal subunit assembly factor), and cell division (*ami*C, N-acetylmuramoyl-L-alanine amidase; *fts*P, cell division protein; *zap*A, cell division protein). This indicated that mild acid treatment influences translation, protein folding, and cell division. SN-10 contains three hub genes encoding the formaldehyde-sensing transcriptional regulator (*frm*ABR), which is associated with the deterioration of fermentation. SN-11 contained three hub genes enriched in ABC transporters (*cys*PU, part of the sulfate/thiosulfate ABC transporter; *ybh*S, part of the probable multidrug ABC transporter permease), indicating the transportation of nutrients and toxins across the membrane under acidic conditions.

## 4. Discussion

The presence of a wide variety of toxic compounds (mainly acids) during industrial fermentation inhibits microbe cell growth, substrate utilization, and product synthesis. The overexpression of the synthetic acid-tolerance module (SC3124) in *E. coli* can enhance cell growth and productivity under mild acid stress [14], which is valuable for industrial applications. In this study, we examined the acid stress response of *E. coli* SC3124 under mildly acidic conditions using a WGCNA model based on transcriptome analysis.

The clustering analysis of the differential gene expression patterns showed that the genes participating in the oxidative phosphorylation and TCA cycle were upregulated in *E. coli* SC3124 at pH 6.0 compared to those at pH 6.8 (Figure 2b). The WGCNA model showed that the SC3124 trait was highly positively correlated with the blue module, which is related to oxidative phosphorylation and the TCA cycle based on GO and KEGG enrichment analyses (Figure 3d and Figure 4). PPI analysis of the hub genes in the blue module revealed eight enriched genes (*nuo*CFGHJLN and *sdh*B) involved in the oxidative phosphorylation and four (*suc*ACD and *sdh*B) involved in the TCA cycle (Figure 5, Table 1). The TCA cycle is an amphibolic pathway, and upregulated enzymes can promote this pathway to generate intermediates for anabolic reactions. Proteomic analysis of *Rhizobium favelukesii* LPU83 showed that three proteins of the TCA cycle (AcnA, SdhD, and SucC) were abundant in acidic conditions [32]. Under aerobic conditions, oxidative phosphorylation is involved in generating a proton motive force (PMF) by coupling metabolic redox reactions with the direct or indirect export of protons from the cell. The increase in these components (via associated gene upregulation) is expected to generate a higher proton export rate under acidic conditions. Through direct proton transport, the cells can actively resist cytoplasmic pH decreases. In many bacteria, selected components of the electron transport chain, including cytochrome *bo* oxidase (*cyo* genes), NADH dehydrogenase I (*nuo* genes), NADH dehydrogenase II (*ndh* genes), and succinate dehydrogenase (*sdh* genes), are upregulated during aerobic growth under mild acid stress (pH 5.0–5.7) conditions [32,33,34].

The accumulation of protons forces the cell to produce increased amounts of ATP and affects the expression of ATP-dependent, membrane-bound transporter proteins of the ABC transporter family, whose members primarily mediate the transport of various nutrients or molecules into cells and toxins or lipids out of cells across membranes through ATP binding and hydrolysis [35]. In the blue module, we found various enriched ABC transporters that are responsible for the transport of different substrates including Dpp, glycine betaine, amino acids (glutathione, lysine, arginine, ornithine, histidine, and cysteine), spermidine, and lipoproteins (Figure 5, Table 1). Many ABC transporters contribute to bile, heat, salt, and acid stress tolerance in bacteria [36,37,38,39]. The Dpp ABC transporter is a common binding-protein-dependent peptide transporter in bacteria [40]. Dpp overexpression improves the survival rates of acid-shock-exposed *Lactococcus lactis* [36]. However, little is known about its acid-stress-related function in *E. coli*. Betaine protects cells from acid stress, and bacterial cells can improve their acid stress tolerance by strengthening the transport of betaine under acidic conditions [41]. Amino acids are key intermediates in both carbon and nitrogen metabolism, and the uptake of amino acids from the external environment is energetically favored by bacteria. Some amino acids are implicated in osmoregulation and pH homeostasis [4,42,43]. *E. coli* has four distinct amino-acid-dependent AR systems: glutamate-, arginine-, lysine-, and ornithine-dependent acid resistance systems, which utilize an amino acid decarboxylase with an externally derived amino acid to consume a proton to generate a byproduct and CO_2_ [4]. Histidine has been found to operate as an intracellular buffer (the pKa of the imidazole moiety is approximately 6.0) in response to acid stress [43]. We hypothesized that the enhanced transport of amino acids could help cells withstand acid stress. The lipoprotein localization ABC transporter is responsible for transporting lipoproteins to the outer membrane [44]. Lipoproteins perform diverse functions in the cell envelope, such as monitoring envelope integrity, stress responses, outer membrane biogenesis, and peptidoglycan synthesis and remodeling [44]. Under acidic conditions, proteins related to the cell envelope are differentially expressed [32,45,46]. For example, bacteria can cope with acid stress by changing the structure and/or composition of peptidoglycans toward more cross-linkages [32,45,46]. Polyamines including putrescine, spermidine, and spermine are necessary for cell proliferation, viability, and stress responses [47]. Genes encoding spermidine and putrescine transporters are upregulated in *Oenococcus oeni* to increase its resistance to an acid-and-ethanol environment. The addition of putrescine and spermidine to the culture medium at physiological concentrations can increase the expression of ROS scavenger genes to help *E. coli* defend against oxidative stress [48]. ROS scavengers are important contributors to the acid-tolerant phenotype of *E. coli* under moderately acidic conditions [7].

The PPI analysis of the hub genes in the blue module showed that SN3- and SN5-containing genes were mainly linked to glutamate (*glt*B, *glt*D), alanine (*ala*A), serine (*ser*C), purine (*pur*FKLRU), and pyrimidine (*pyr*C) biosynthesis processes (Figure 5, Table 1). Proteomic and transcriptomic analyses of several bacteria have shown that amino acid metabolism is altered under acid stress [12,32,43,45,46,49], indicating that amino acid metabolism plays an important role in acid stress tolerance. As mentioned above, some amino acids participate in intracellular pH homeostasis [3,4]. In bacteria, glutamate metabolism plays an important role in the response to acid stress via the GDAR system [42]. However, no notable alterations were observed in the expression of genes related to the GDAR system in *E. coli* SC3124 at pH 6.0. Amino acid metabolism is the main proteome resource in cells. Enhanced amino acid metabolism activity likely satisfies the requirements for energy and proteome resources for related metabolic and various cellular processes in a low-pH environment [45]. Oxidative phosphorylation, the TCA cycle, cofactor and prosthetic group biosynthesis, glycolysis, and gluconeogenesis are the main processes requiring additional proteome resources under acid stress and hence are the major drivers of the upregulation of amino acid biosynthesis [12]. The purine and pyrimidine biosynthetic pathways were linked to the “alanine, aspartate, and glutamate metabolism” pathway by carbamoyl phosphate. Changes in amino acid metabolism may affect purine and pyrimidine biosynthetic processes.

**Figure 6:**
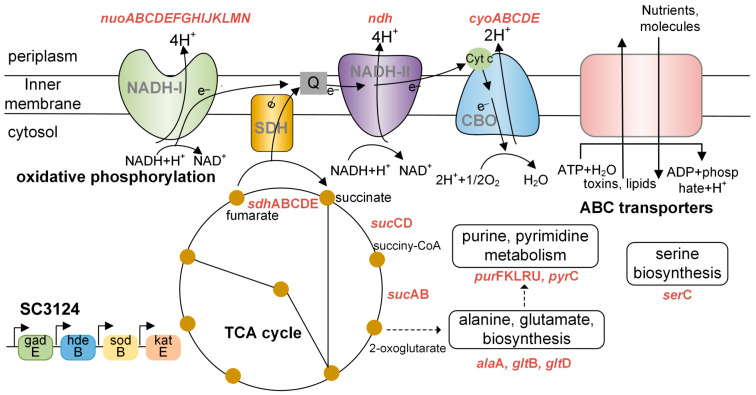
Diagram of metabolic changes in response to mild acid stress in *E. coli* SC3124. Abbreviations: CBO, cytochrome *bo* oxidase; NADH-I, NADH dehydrogenase I; NADH-II, NADH dehydrogenase II; SDH, succinate dehydrogenase; Q, quinone; Cytc, cytochrome; SC3124, synthetic acid-tolerance module. The upregulated and enriched genes identified in the PPI analysis of the blue module are shown in red.

The acid stress response mechanism is complex in *E. coli*, which requires the coordination of a range of metabolic, physiological, and proton-consuming AR systems [4]. Physiological adaptations include membrane modifications and outer membrane porins to reduce proton influx, as well as periplasmic and cytoplasmic chaperones to manage the effects of acid damage [1,3,4]. Under aerobic conditions, metabolic acid stress tolerance systems couple proton efflux to induce energy generation by selecting components of the electron transport chain [4,33,34]. Proton-consuming AR systems comprise four amino-acid-dependent AR systems: GDAR, LDAR, ADAR, and ODAR [4,50]. Generally, metabolic and physiological changes, as well as the LDAR and ODAR systems, are activated in *E. coli* to resist mild and moderate acid stress [4]. The SC3124 trait was highly positively associated with the blue module, which is linked to oxidative phosphorylation, the TCA cycle, ABC transporters, amino acids, and purine metabolism, suggesting that overexpression of the synthetic acid-tolerance module in *E. coli* mainly caused metabolic changes to resist acid stress (Figure 6), which belongs to the acid tolerance response system to mild acid stress. The enhanced growth under mild acid stress could be attributed to the upregulation of metabolism-associated genes. However, the underlying molecular mechanism of direct or indirect SC3124-induced metabolic changes in *E. coli* in response to mild acid stress requires further investigation.

## 5. Conclusions

In summary, the synthetic acid-tolerance module confers mild acid stress resistance in *E. coli* mainly through metabolic changes, including an increase in the oxidative phosphorylation, TCA cycle, ABC transporter, amino acid metabolism, and purine metabolism pathways. This work provides novel insights into generating an acid-tolerant microbial chassis for biotechnology applications.

## Figures and Tables

**Table 1:** Enrichment of the protein–protein interaction subnetworks in the blue module.

Subnetwork	Gene	Product	GO Pathway ^1^	KEGG Pathway ^1^
SN.1	*dppF*	dipeptide ABC transporter ATP binding subunit DppF	Transporter activity; transmembrane transporter activity; nitrogen compound transport; establishment of localization; amino acid transport; carboxylic acid transport; monoatomic cation transmembrane transport; monoatomic ion transmembrane transport.	ABC transporters
	*lolE*	lipoprotein release complex—inner membrane subunit		
	*lolD*	lipoprotein release complex—ATP binding subunit		
	*artQ*	L-arginine ABC transporter membrane subunit ArtQ		
	*dppD*	dipeptide ABC transporter ATP binding subunit DppD		
	*artI*	putative ABC transporter periplasmic binding protein ArtI		
	*proX*	glycine betaine ABC transporter periplasmic binding protein ProX		
	*cydC*	glutathione/L-cysteine ABC exporter subunit CydC		
	*ydcS*	putative ABC transporter periplasmic binding protein/polyhydroxybutyrate synthase		
	*dppB*	dipeptide ABC transporter membrane subunit DppB		
	*potC*	spermidine preferential ABC transporter membrane subunit PotC		
	*hisM*	lysine, arginine, ornithine ABC transporter/histidine ABC transporter, membrane subunit HisM		
	*lolC*	lipoprotein release complex—inner membrane subunit		
	*potB*	spermidine preferential ABC transporter membrane subunit PotB		
	*hisQ*	lysine, arginine, ornithine ABC transporter/histidine ABC transporter, membrane subunit HisQ		
	*osmF*	glycine betaine ABC transporter periplasmic binding protein OsmF		
	*dppC*	dipeptide ABC transporter membrane subunit DppC		
	*dppA*	dipeptide ABC transporter periplasmic binding protein		
SN.2	*nuoJ*	NADH:quinone oxidoreductase subunit J	NAD(P)H dehydrogenase (quinone) activity; oxidoreductase activity, acting on NAD(P)H; electron transfer activity.	Oxidative phosphorylation; citrate cycle (TCA cycle).
	*sucD*	succinyl-CoA synthetase subunit alpha		
	*nuoC*	NADH:quinone oxidoreductase subunit CD		
	*nuoN*	NADH:quinone oxidoreductase subunit N		
	*nuoF*	NADH:quinone oxidoreductase subunit F		
	*sucB*	dihydrolipoyltranssuccinylase		
	*nuoH*	NADH:quinone oxidoreductase subunit H		
	*nuoG*	NADH:quinone oxidoreductase subunit G		
	*sucA*	subunit of E1(0) component of 2-oxoglutarate dehydrogenase		
	*nuoL*	NADH:quinone oxidoreductase subunit L		
	*sdhB*	succinate:quinone oxidoreductase, iron-sulfur cluster binding protein		
SN.3	*pxpC*	5-oxoprolinase component C	Purine biosynthesis; pyrimidine nucleotide biosynthesis; glutamate biosynthesis; 5-oxoprolinase (ATP-hydrolyzing) activity; toxin biosynthesis.	Purine, pyrimidine metabolism; glutathione metabolism; alanine, aspartate and glutamate metabolism.
	*purR*	DNA-binding transcriptional repressor PurR		
	*pyrC*	dihydroorotase		
	*purF*	amidophosphoribosyltransferase		
	*pxpA*	5-oxoprolinase component A		
	*purK*	5-(carboxyamino)imidazole ribonucleotide synthase		
	*purL*	phosphoribosylformylglycinamide synthetase		
	*cvpA*	colicin V production protein		
	*gltD*	glutamate synthase subunit GltD		
SN.4	*epmC*	EF-P-Lys34 hydroxylase	Not available.	Not available.
	*yfcA*	putative transporter YfcA		
	*yfcL*	PF08891 family protein YfcL		
	*mepA*	peptidoglycan DD-endopeptidase/peptidoglycan LD-endopeptidase		
SN.5	*alaA*	glutamate--pyruvate aminotransferase AlaA	L-alanine biosynthesis; glutamate biosynthesis; serine biosynthesis; purine nucleotide biosynthesis; plasma membrane.	Alanine, aspartate and glutamate metabolism; glycine, serine and threonine metabolism; purine metabolism.
	*ycdX*	zinc-binding phosphatase YcdX		
	*serC*	phosphoserine/phosphohydroxythreonine aminotransferase		
	*ghrA*	glyoxylate/hydroxypyruvate reductase A		
	*purU*	formyltetrahydrofolate deformylase		
	*ycdY*	chaperone protein YcdY		
	*gltB*	glutamate synthase subunit GltB		
	*ycdZ*	inner membrane protein YcdZ		
SN.6	*prfA*	peptide chain release factor RF1	Not available.	Not available.
	*ispE*	4-(cytidine 5′-diphospho)-2-C-methyl-D-erythritol kinase		
	*hemA*	glutamyl-tRNA reductase		
SN.7	*folC*	bifunctional folylpolyglutamate synthetase/dihydrofolate synthetase	Folic acid biosynthetic process.	Folate biosynthesis.
	*folP*	dihydropteroate synthase		
	*folX*	dihydroneopterin triphosphate 2′-epimerase		
SN.8	*yegS*	lipid kinase YegS	Not available	Not available
	*ymgG*	PF13488 family protein YmgG		
	*ymgD*	PF16456 family protein YmgD		

^1^ Genes in each subnet were enriched in GO and KEGG databases, and the items with minimum FDR (<0.05) are listed.

**Table 2:** Enrichment of the protein–protein interaction subnetworks in the pink module.

Subnetwork	Gene	Product	GO Pathway ^1^	KEGG Pathway ^1^
SN.9	*yidC*	membrane protein insertase YidC	Not available	Not available
	*gltX*	glutamate-tRNA ligase		
	*rnpA*	RNase P protein component		
	*bipA*	50S ribosomal subunit assembly factor BipA		
SN.10	*frmR*	DNA-binding transcriptional repressor FrmR	Formaldehyde catabolic process	Microbial metabolism in diverse environments
	*frmB*	S-formylglutathione hydrolase FrmB		
	*frmA*	S-(hydroxymethyl)glutathione dehydrogenase		
SN.11	*cysP*	thiosulfate/sulfate ABC transporter periplasmic binding protein CysP	Thiosulfate/sulfate transporter; sulfur metabolism; xenobiotic transmembrane transporter	Sulfur metabolism; ABC transporters
	*cysU*	sulfate/thiosulfate ABC transporter inner membrane subunit CysU		
	*ybhS*	probable multidrug ABC transporter permease		
SN.12	*amiC*	N-acetylmuramoyl-L-alanine amidase C	Not available	Cationic antimicrobial peptide (CAMP) resistance

^1^ Genes in each subnet were enriched in GO and KEGG databases, and the items with minimum FDR (< 0.05) are listed.

## Data Availability

The raw sequence data were deposited in the NCBI Sequence Read Archive Database under accession number PRJNA1036286.

## Supplementary Materials

The following supporting information can be downloaded at: https://www.mdpi.com/article/10.3390/microorganisms12081565/s1, Figure S1: Scatter plots of eigengenes in selected modules (correction coefficient > 0.55 and *p* ≤ 0.01). X-axis represents the module membership value of each gene within the respective module. Y-axis depicts a measure of significance for each gene’s association with acidity-related traits. The colors in each subplot correspond to the respective module names. These colors facilitate the visual distinction between modules and allow for easy tracking of eigengene profiles across different gene groups. Table S1: Gene significance and module membership in WGCNA modules.
