# Supplementary figures and images for: Characterization of Mild Acid Stress Response in an Engineered Acid-Tolerant Escherichia coli Strain
Source: Microorganisms. 2024 Jul 31;12(8):1565. doi: 10.3390/microorganisms12081565 (PMC11356199; doi:10.3390/microorganisms12081565)

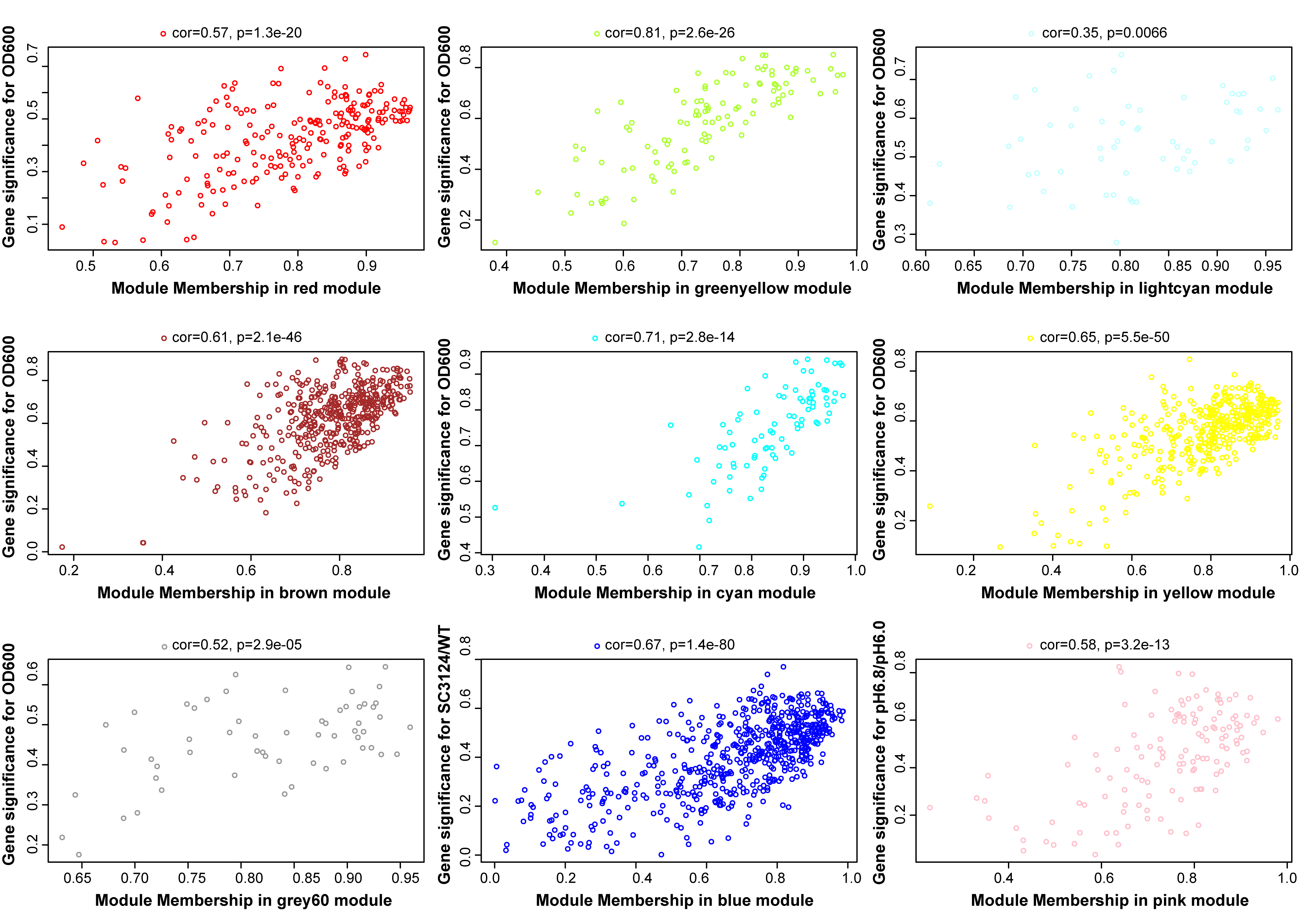

Supplement: Supplementary file 1 [file microorganisms-12-01565-s001.zip › Figure S1.tif]
